# Biological Effects of EF24, a Curcumin Derivative, Alone or Combined with Mitotane in Adrenocortical Tumor Cell Lines

**DOI:** 10.3390/molecules24122202

**Published:** 2019-06-12

**Authors:** Loris Bertazza, Susi Barollo, Maria Elena Mari, Irene Faccio, Maira Zorzan, Marco Redaelli, Beatrice Rubin, Decio Armanini, Caterina Mian, Raffaele Pezzani

**Affiliations:** 1 Endocrinology Unit, Department of Medicine (DIMED), University of Padova, via Ospedale 105, 35128 Padova, Italy; loris.bertazza@unipd.it (L.B.); susi.barollo@unipd.it (S.B.); mariaelena.mari@studenti.unipd.it (M.E.M.); irene.faccio1993@gmail.com (I.F.); maira.zorzan@vimset.it (M.Z.); rubin.beatrice@gmail.com (B.R.); decio.armanini@unipd.it (D.A.); caterina.mian@unipd.it (C.M.); 2 AIROB, Associazione Italiana per la Ricerca Oncologica di Base, 3520128 Padova, Italy; marcoredaelli@email.it; 3 Venetian Institute for Molecular Science and Experimental Technologies, VIMSET. Pz Milani 4, 30010 Campolongo Maggiore, Italy

**Keywords:** EF24, curcumin, adrenocortical, preclinical cell model

## Abstract

Background: Curcumin has numerous properties and is used in many preclinical conditions, including cancer. It has low bioavailability, while its derivative EF24 shows enhanced solubility. However, its effects have never been explored in adrenocortical tumor cell models. The efficacy of EF24 alone or combined with mitotane (reference drug for adrenocortical cancer) was evaluated in two adrenocortical tumor cell lines, SW13 and H295R. Method and Results: EF24 reduced cell viability with an IC50 (half maximal inhibitory concentration) of 6.5 ± 2.4 μM and 4.9 ± 2.8 μM for SW13 and H295R cells, respectively. Combination index (EF24 associated with mitotane) suggested an additivity effect in both cell lines. Cell cycle analysis revealed an increase in subG0/G1 phase, while motility assay showed a decrease in migratory cell capacity, and similarly, clonogenic assay indicated that EF24 could reduce colony numbers. Furthermore, Wnt/β-catenin, NF-κB, MAPK, and PI3k/Akt pathways were modulated by Western blot analysis when treating cells with EF24 alone or combined with mitotane. In addition, intracellular reactive oxygen species levels increased in both cell lines. Conclusion: This work analyzed EF24 in adrenocortical tumor cell lines for the first time. These results suggest that EF24 could potentially impact on adrenocortical tumors, laying the foundation for further research in animal models.

## 1. Introduction

**Figure 1:**
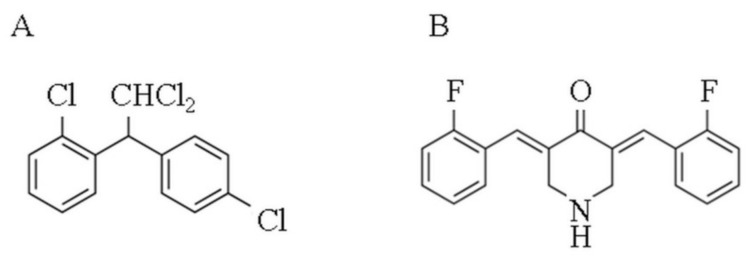
Chemical structure of the two compounds used in subsequent experiments. (**A**) mitotane (1-(2-Chlorophenyl)-1-(4-chlorophenyl)-2,2-dichloroethane,1-Chloro-2-[2,2-dichloro-1-(4-chlorophenyl)ethyl]-benzene); (**B**) and EF24 ((3E,5E)-3,5-bis[(2-fluorophenyl)methylene]-4-piperidinone).

Adrenocortical tumors (ACT) are common diseases with a prevalence of 3–10% in the general population and can be categorized into adrenocortical adenomas (ACA), which is more frequent, and adrenocortical carcinoma (ACC), which is very rare and has an incidence of 1 to 2 per million per year [1]. ACC has a five-year survival rate of approximately 20–35% and can frequently metastasize; for these reasons, the prognosis is often poor [2]. Moreover, the majority of patients are diagnosed with advanced disease, which does not permit an actual ACC treatment. However ACC management is essentially based on surgical removal of the mass, which is the only complete curative option [3]. When at the diagnosis, a metastasis is detected, clinicians can use mitotane, the reference adrenotoxic agent approved for ACC, in addition to cytotoxic drugs that should be supplemented in case of disease progression (Figure 1A) [3]. Nonetheless a wide proportion of ACC patients still worsen in condition [4]. Numerous works have been attempted to understand its pathophysiology. Recently, different Omics analyses have deepened the genetic and epigenetic background of ACC [5,6,7]. Although our knowledge of tumorigenesis has concretely improved in the last decade, pharmacological treatments are still insufficient. The lack of effective chemotherapeutic drugs drive researchers to explore novel compounds that are potentially useful in the treatment of this neoplasia. Preclinical animal and cellular studies can contribute to the development of new anticancer agents.

Curcumin is a natural product extracted from *Curcuma longa* L. with numerous properties used since ancient times in Chinese and Indian traditional medicines [8]. Curcumin has been also used in cancer, and several preclinical and clinical works have reported its efficacy [9,10]. Even with recognized and valuable effects, curcumin has poor bioavailability and solubility, which has led researchers to discover a more soluble derivative with similar safety profiles and enhanced anticancer activity, such as EF24 (Figure 1B) [11,12,13].

Given these premises, this in vitro work analyzed the effects of EF24 alone or in combination with mitotane in adrenocortical tumor cell models, SW13 and H295R cells, for the first time. The effects were examined by cytotoxic cell assays, motility assays, clonogenic assays, cell cycle analysis, cell morphology, signaling pathway modulation, and intracellular reactive oxygen species production.

## 2. Results

### 2.1. Cell Viability Assays, Combination Index, and Drug Synergism

**Figure 2:**
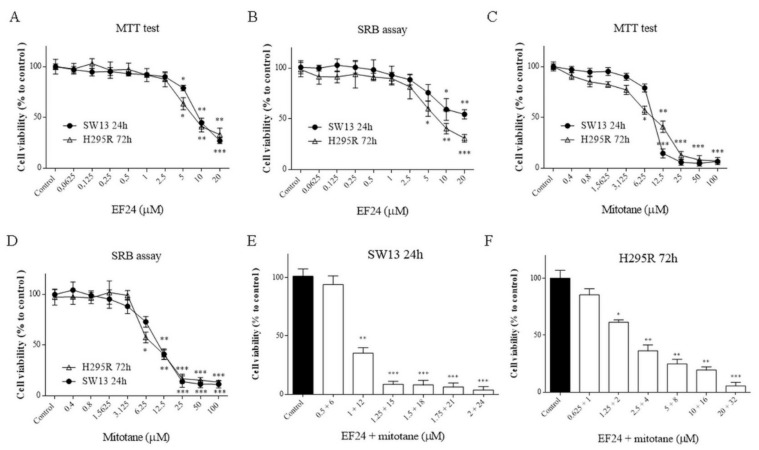
MTT and SRB assay for SW13 and H295R cells treated for 24 h and 72 h. (**A**) MTT test for EF24; (**B**) SRB assay for EF24; (**C**) MTT test for mitotane; (**D**) SRB assay for mitotane; (**E**) MTT test for combination index calculation in SW13 at 24 h; (**F**) MTT test for combination index calculation in H295R at 72 h. Different drug concentrations were used following a series of CI values generated by the CompuSyn 3.0.1 program. Experiments were performed in quadruplicate and repeated three times. Treatment vs. control: * *p* < 0.05, ** *p* < 0.01, *** *p* < 0.001.

The effects of EF24 were first analyzed by cell viability. By the MTT assay, we showed that the IC50 of EF24 was 6.5 ± 2.4 μM and 4.9 ± 2.8 μM for SW13 at 24 h and H295R cells at 72 h, respectively (Figure 2A). By SRB (sulforhodamine B) assay we revealed that the IC50 of EF24 was 5.3 ± 2.7 μM and 9.1 ± 3.1 μM for SW13 and H295R cells, respectively (Figure 2B). The effects of the compound suggest a dose-dependent effect. After these experiments, we decided to use the IC50 concentration for EF24 in most of subsequent experiments (if not otherwise indicated): 6.5 μM for SW13 cells and 5 μM for H295R cells. Similarly, we calculated IC50 for mitotane in both cell lines: 8.1 ± 3.2 µM for SW13 at 24 h and 10.6 ± 2.3 µM for H295R at 72h (Figure 2C,D). Consequently, we decided to use the IC50 concentration for mitotane in subsequent experiments: 8 μM for SW13 cells and 10 μM for H295R cells. Moreover, the calculated CI (combination index) for EF24 associated with mitotane, the reference drug for ACC, was 1.1 in SW13 cells and 0.9 in H295R.

### 2.2. Cell Cycle Analysis

**Figure 3:**
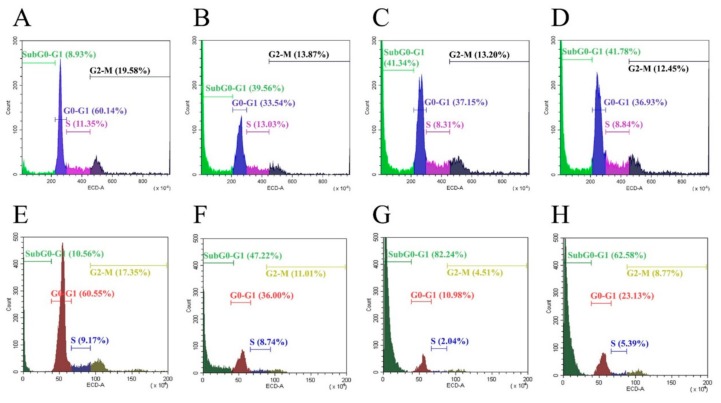
Representative cell cycle analyses. SW13 cells treated at 24 h: (**A**) Control; (**B**) EF24 6.5 μM; (**C**) EF24+mitotane; (**D**) mitotane 8 μM. H295R cells treated at 72 h: (**E**) Control; (**F**) EF24 5 μM; (**G**) EF24 + mitotane; (**H**) mitotane 10 μM. Experiments were performed in triplicate.

Cell cycle analysis was evaluated in SW13 and H295R cells in order to find any modulation of cell cycle distribution. An increase in subG0/G1 phase compared to control was observed in all treatments (EF24 alone or combined to mitotane) (Figure 3A–H). A concomitant decrease of G0/G1 phase and reduction of G2/M phases were observed in all cell experiments.

### 2.3. Motility Assay (Wound Healing Assay)

**Figure 4:**
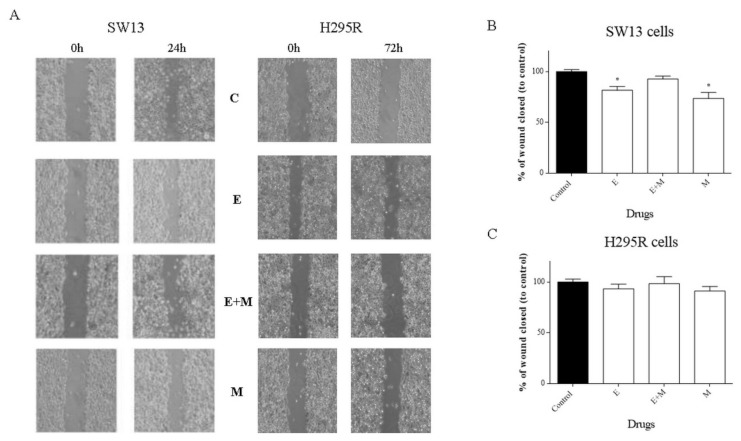
Cell motility assay for SW13 and H295R cells treated with EF24, mitotane, and their combination at 24 h and 72 h, respectively. (**A**) Representative images of the wound healing assay (C = control, E = EF24, M = mitotane, E + M = EF24 + mitotane). (**B,C**) Quantification of cell motility for SW13 and H295R cells. Results are expressed as the percentage reduction of the initial scratch compared with the corresponding untreated cells. Data are shown as the means of nine measurements. Experiments were performed in triplicate. Treatment vs. control: * *p* < 0.05.

Motility assays (wound healing assay) can examine the capacity of cells to cover an empty space. It is known that ACC can frequently metastasize: We showed a significant reduction in cell motility after treatments with EF24 and mitotane in SW13 cells (Figure 4A,B). In contrast, in H295R cells, the treatments did not show an effect on cell motility (Figure 4A–C).

### 2.4. Assessment of Cell Morphology by Wright’s Staining 

**Figure 5:**
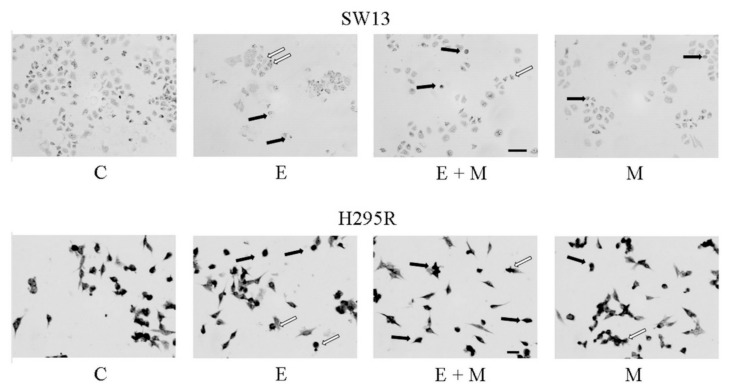
Cell morphology evaluated by Wright’s staining method after treatment with EF24, mitotane, and their combination in SW13 and H295R cells treated at 24 h and 72 h, respectively. The arrows show apoptotic (white) or necrotic cells (black). (C = control, E = EF24, M = mitotane, E + M = EF24 + mitotane).

Any treatment in a living system such as a cell can potentially modify its shape or the morphology. With Wright’s staining, we showed the presence of cell death in both cell lines, specified by an arrow in Figure 5.

### 2.5. Clonogenic Cell Survival Assay

**Figure 6:**
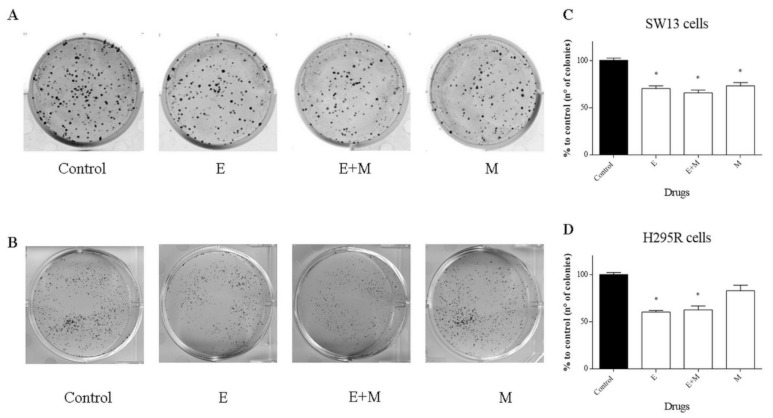
Representative clonogenic assay in SW13 and H295R cells treated with EF24, mitotane, and their combination at 24 h and 72 h, respectively. (**A**) SW13 cells. (**B**) H295R cells. (**C**,**D**) Statistical analysis (C = control, E = EF24, M = mitotane, E + M = EF24 + mitotane). Treatment vs. control: * *p* < 0.05. Each experiment was performed in triplicate and repeated twice.

The clonogenic survival assay is a test based on the ability of a single cell to grow into a colony, which is a hallmark of cancer and can analyze stemness-like activity of the cells. The effects of EF24 and its combination in tumor cells showed a decrease in colony number in all treatments and in both cell lines (Figure 6A–C).

### 2.6. Western Blot Analysis 

**Figure 7:**
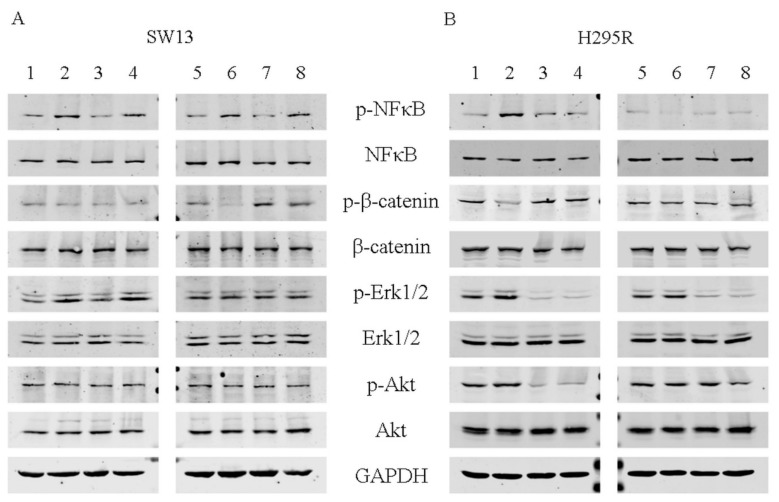
Representative Western blot analyses for adrenocortical tumor cells. (**A**) SW13 cells; (**B**) H295R cells; 1 and 5 = untreated control; 2 and 6 = EF24 at 6.5 μM for SW13 and 5 μM for H295R cells; 3 and 7 = mitotane 8 μM for SW13 cells and 10 μM for H295R cells; 4 and 8 = EF24 + mitotane. Experiments were performed in triplicate.

We then analyzed the effects of EF24 on cell signaling (Figure 7 and Figure S1). We showed that phospho-NF-κB was augmented by EF24 at 5 h in both cell lines and at 72 h in SW13 cells only (Figure 7A,B). Additionally, the combination of EF24 and mitotane induced an increase of phospho-NF-κB expression in SW13 cells at 5 h and 72 h, while in H295R cells, no similar result was appreciable (Figure 7A,B). Mitotane alone seemed to not alter phospho-NF-κB in both cell lines. Furthermore, phospho-β-catenin was reduced after 5 h of EF24 treatment in H295R cells and after 72 h in SW13 cells. Phospho-Erk1/2 was increased in SW13 cells at 5 h by EF24 treatment or its combination with mitotane. In contrast, in H295R cells, phospho-Erk1/2 was reduced by mitotane treatment (alone or in association with EF24) at both 5 h and 72 h (Figure 7A,B). Phospho-Akt was unaltered in SW13 cells, while it was reduced in H295R cells at 5 h by mitotane treatment (alone or in association with EF24).

### 2.7. Intracellular Reactive Oxygen Species Levels (DCFH-DA Assay)

**Figure 8:**
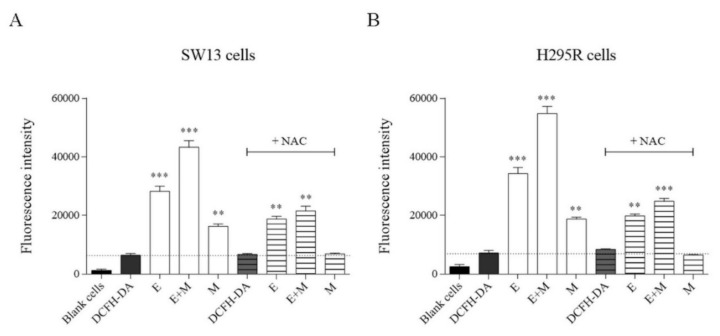
Histograms of intracellular levels of reactive oxygen species in SW13 (**A**) and H295R (**B**) cells after treatment with EF24, mitotane, and their combination. DCFH-DA = 2′,7′-dichlorofluorescein diacetate. NAC = N-acetil-cysteine. E = EF24. M = mitotane. E + M = EF24 + mitotane. Treatment vs. control: ** *p* < 0.01, *** *p* < 0.001. Experiments were performed in triplicate and repeated twice.

Reactive oxygen species (ROS) are the main contributors of oxidative stress, a phenomenon that can lead to different diseases. We investigated whether EF24 or its combination with mitotane could increase ROS levels (Figure 8 and Figure S2). We showed that all tested compounds augmented ROS levels in both cell lines (Figure 8A,B). In particular, EF24 induced a major ROS increase compared to mitotane, while their combination (EF24 + mitotane) further enhanced ROS levels in both cell lines. The use of NAC partially recovered the ability of tumor cells to withstand the oxidative stress promoted by the treatments (Figure 8). 

## 3. Discussion

This research work analyzed, for the first time, the use of EF24, a curcumin derivative with increased availability, in two adrenocortical tumor cell lines, SW13 and H295R cells, the only adrenocortical tumors available for studying [14]. EF24 was first described and discovered by the research of Adams et al. in 2004, and from that year, numerous works were published investigating the effects of EF24 in preclinical models [12]. For an updated and comprehensive review of EF24, the reader can refer to the work of He et al. [13].

First, we investigated the effects of EF24 in a cell viability assay. As shown in Figure 2, EF24 decreased cell viability in a time- and concentration-dependent way in both cell lines, with an IC50 of 6.5 μM for SW13 cells and 5 μM for H295R cells. Moreover, the CI obtained by subsequent experiments combining EF24 and mitotane (the reference drug for ACC) demonstrated an additive effect: We considered CI = 1 ± 0.19 as additivity, even if from a technical point of view, 1.1 in SW13 cells means antagonism and 0.9 in H295R cells means synergism (Figure 2E,F). Similar results were found in ovarian carcinoma cells (SK-OV-3 and IGROV1), where EF24 induced a time- and dose-dependent suppression of cell growth and viability [15]. Considering cell cycle analysis, and in contrast from the works on liver and colon cell cancers that showed a G2/M phase arrest [16,17], we showed the presence of cell death in both cell lines (subG0/G1 augmentation) (Figure 3). This result supported a substantially unaltered cell cycle distribution in adrenocortical cell lines treated by EF24. Moreover, it emphasizes how EF24 can induce a strong cell death mechanism, as suggested by cell viability data and cell morphology (Figure 5). In addition, this compound can potentially act on the cell migration ability of SW13 tumor cells: The results showed a reduction in the capacity of cells in covering an empty space (reduction in wound healing recovering), an issue strictly related to metastasis. Similarly, in ovarian and hepatocellular carcinoma cell lines, EF24 could reduce cell migration aptitude [18,19]. However, only SW13 cells seem to be affected, probably because they have a different genetic background with an elevated doubling time if compared to H295R cells. In support of a potential role of EF24 in colony number reduction, the clonogenic assay showed that the compound could limit the stemness-like activity of tumor cell models (Figure 6), which is in line with previous studies on lung cancer cells [20].

We already demonstrated that PI3k/Akt, MAPK, and Wnt/β-catenin pathways are altered in adrenocortical tumor cell lines [21,22]. EF24 can potentially act on these fundamental cell signaling pathways. Nonetheless, in H295R cells, only phospho-β-catenin was reduced after 5 h (Figure 7A,B). The switching off of β-catenin can impact on ACC cell survival, as more than 60% of this neoplasm showed its nuclear accumulation in a previous preclinical work [22]. As expected, only mitotane alone or combined with EF24 decreased the reactivity of Erk1/2 and Akt. Of note, phospho-NF-κB was augmented by EF24 at 5 h in both cell lines and at 72 h in H295R cells (Figure 7A,B); similar results have already been reported, underlining that small doses of curcumin could enhance proliferation and survival [23,24] and could possibly potentiate cell death by DNA damage or oxidative stress [25,26,27]. On the same line, we showed an increase in phosphorylation of Erk1/2 at 5 h in SW13 cells (by EF24 alone or combined), suggesting that under certain circumstances, Erk1/2 can have pro-apoptotic functions, as it is the most important balance between pro- and anti-proliferative signals (Figure 3 and Figure S1), which was already reported by Thomas et al. in 2010, where EF24 was shown to significantly induce the upregulation of Erk1/2, JNK, and p38 (MAPK pathway) [20,28,29]. Interestingly, when treating SW13 cells with EF24, phospho-β-catenin was reduced, while phospho-NF-κB was increased, suggesting a plausible crosstalk between Wnt/β-catenin and inflammation pathways as already reported. However, other research is certainly needed to demonstrate this hypothesis [30].

Moreover, an increase of intracellular ROS levels was observed (Figure 8 and Figure S2). This accumulation of ROS was already observed in human breast, prostate, and gastric cancer cells [31,32], but never in ACC. ROS are a normal by-product of numerous cellular processes, such as mitochondrial metabolism and protein folding [33]. Compared to normal cells, cancer cells have intrinsically higher levels of ROS and are under oxidative stress due to an imbalanced redox status [33,34]. Both SW13 and H295R showed a significant increase of ROS levels with EF24, mitotane, or their combination. This accumulation could potentially explain the effective anti-tumor role of EF24 in different cell models [33,34,35]. Similarly, EF24 was able to generate ROS production in MDA-MB-231 (human breast cancer) cells; DU-145 (human prostate cancer) cells; SGC-7901, BGC-823, KATO III (human gastric cancer) cells; and HCT-116, SW-620, and HT-29 (human colon cancer) cells [16,31,32].

## 4. Materials and Methods

### 4.1. Materials and Reagents

Dimethyl sulfoxide (DMSO), fetal bovine serum (FBS), 3-[4,5-dimethylthiazol-2-yl]-2,5 diphenyl tetrazolium bromide (MTT), propidium iodide (PI), mitotane (1,1-(dichlorodiphenyl)-2,2-dichloroethane (o,p’-DDD)), and EF24 were purchased from Sigma Aldrich, Italy. DMEM-F12, 0.05% trypsin-EDTA, insulin, transferrin, selenium, and antibiotics were from ThermoFisher Scientific, Italy. The primary antibodies used were Akt (cod. 9272), phospho-Akt (Ser473) (cod. 9271), Erk1/2 (cod. 4695), phospho-Erk1/2 (Thr202/Tyr204) (cod. 4370), NF-κB p65 (cod.8242), and phospho-NF-κB p65 (cod.3033), all from Cell Signaling Technology, USA; GAPDH (cod.8245), β-catenin (cod.32572), and phospho-β-catenin (cod. 81305) from Abcam, UK. The secondary antibodies used were IRDye 800 CW anti-mouse and IRDye 680 RD anti-rabbit (LiCor, Lincoln, OR, USA).

### 4.2. Cell Cultures and Maintenance

H295R and SW13 adrenocortical tumor cell lines were obtained from the American Type Culture Collection (ATCC, Rockwille, MD, USA). H295R cells were derived from a female patient diagnosed with ACC and the cells can secrete mineral corticoids, glucocorticoids and adrenal androgens. SW13 cells were a metastatic depot in the adrenal cortex of a 55-year-old female patient derived from a primary small cell lung carcinoma. This strain produces no steroid [14]. Moreover, SW13 cells have a doubling time of about 24 h, while H295R cells show >48–72 h doubling time; this difference has been considered in cell experiments. All cells were cultured as previously described [21].

### 4.3. Cell Viability Assays, Combination Index, and Drug Synergism

Cells were plated in 96-well plates at a density of 5 × 10^3^ cells/well in supplemented medium with or without the tested compounds. SW13 cells were treated at 24 h (and 72 h), while H295R cells were treated at 72 h (and 24 h). EF24 was used at 0.0625, 0.125, 0.25, 0.5, 1, 2.5, 5, 10, and 20 μM, and mitotane was used at 0.4, 0.8, 1.5625, 3.125, 6.25, 12.5, 25, 50, and 100 μM. Both EF24 and mitotane were tested by MTT assay and SRB assay. The maximum drug effect was experimentally observed at the endpoint, and thus the IC50 value after 24–72 h (depending on cell doubling time) of treatment was determined. All experiments were performed in quadruplicate and repeated 3 times.

The combination index (CI) values were calculated using the CompuSyn 3.0.1 program [36]. Based on specific dose–response curves, using the MTT assay for cells treated with compounds alone or in combination at a constant ratio, a series of CI values were generated over a range of levels of growth inhibition from 5% to 95% of the fraction affected. The values at 50% growth inhibition are presented for regimen combination. Synergism, additive effect, and antagonism are defined as CI < 1, CI = 1, and CI > 1, respectively [35].

### 4.4. Cell Cycle Analysis

Cells were plated into 25 cm^2^ flasks at a density of 1 × 10^6^ cells and were treated with EF24, mitotane or the combination for 24 h (SW13 cells) or 72 h (H295R cells), as previously established [37]. IC50 was used for the treatments. Cells were detached by trypsin-EDTA, re-suspended in ice-cold PBS (Phosphate Buffered Saline), and fixed in 70% ice-cold ethanol followed by an overnight incubation at −20 °C. After washing, cells were stained with PI solution (50 μg/mL PI, 10 μg/mL RNaseA) and incubated for 1 h at 37 °C in the dark. Cell cycle analysis was performed in triplicates and data were assessed by a CytoFLEX Beckman Counter. The experiments were performed in triplicate.

### 4.5. Motility Assay (Wound Healing Assay)

Cells were seeded in 6-well plates at 2 × 10^6^ cells/well and treated for 24 h (SW13 cells) and 72 h (H295R cells) with the compounds and their combination at IC50 doses. The medium was renewed, and a scratched wound was created using a pipette tip. Cells migrated into the wound surface, and the average distance of the migrating cells was determined under an inverted microscope (40×) at 0 h and checked every day until the end of the experiment (after 1 week and 2 weeks for SW13 and H295R cells, respectively). The experiments were performed in triplicate and repeated 3 times.

### 4.6. Assessment of Cell Morphology by Wright’s Staining 

SW13 and H295R cells (5×10^4^ cells/well in 24-well plates) were grown on coverslips for 48 h and then treated for 24 h (SW13 cells) and 72 h (H295R cells) with the compounds and their combination at IC50 doses. Treated cells were fixed in methanol for 5 min, stained with Wright’s stain, and observed under a light microscope for evaluation of cell morphology as previously described [37]. At least 600 cells were counted for every experiment in 10 different fields, and each experiment was repeated twice.

### 4.7. Clonogenic Cell Survival Assay

Cells were seeded in 6-well plates at a low density (1000 cells per well), incubated overnight in 0.1% FBS, and then treated with EF24, mitotane, or their combination at IC50 concentrations for 24 h (SW13 cells) and 72 h (H295R cells). Then, the cell medium was replaced with free medium and cell-cultured for 1 week (SW13 cells) or 2 weeks (H295R cells). Cells were then fixed and stained with crystal violet. Only colonies of >50 cells were counted. Each experiment was performed in triplicate and repeated 2 times.

### 4.8. Western Blot Analysis 

SW13 and H295R cells were treated with IC50 doses for 5 h and for 24 h (SW13) or 72 h (H295R). Protein was extracted and electro-blotted onto nitrocellulose membranes as previously described [38]. Primary antibodies were incubated overnight, and then secondary antibodies were added for 1 h with anti-mouse and anti-rabbit (1:800) secondary IRDye. Membranes were scanned with the Odyssey CLX system (LI-COR BioSciences, Milan, Italy) equipped with infrared light technology for detection. Signal intensity was quantified with Image Studio™ software (Version 4.0, LI-COR) following manufacturer’s instructions. The experiments were performed in triplicate.

### 4.9. Intracellular Reactive Oxygen Species (ROS) Levels (DCFH-DA Assay)

SW13 and H295R cells (3 × 10^5^ per well in a six-well plates) were incubated with EF24, mitotane, and their combination for 2 h at IC50 doses. N-acetil-cysteine (NAC) was used as the internal control. Thirty minutes before the end of the treatment, 2′,7′-dichlorofluorescein diacetate (DCFH-DA; Sigma-Aldrich, Milano, Italy) was added to each sample at a final concentration of 5 μM. Cells were then harvested, and one-half of them was used for flow cytometry (CytoFLEX Analyzer, Beckman Counter, CA, USA), whereas the fluorescence of the remaining cells was quantified with a plate reader (Victor-X3 multilabel counter, PerkinElmer, Finland). Flow cytometry data were acquired and analyzed using the CytExpert software (Beckman Coulter, CA, USA). For oxidative stress induction, as an internal control, cells were treated with 10 μM H_2_O_2_ for 2 h in complete medium (data not shown), and at least 10,000 events were collected for each sample. The experiments were performed in triplicate and repeated 2 times.

### 4.10. Statistical Analysis

Statistical analysis was performed using both MedCalc software (version 11.2.1.0) and GraphPad Prism (version 6). A *p*-value of <0.05 was considered statistically significant. The Kolmogorov–Smirnov test was used to evaluate the normal distribution of each numeric parameter. Data comparisons were performed using the two-tailed Student’s t-test and Kruskal–Wallis analysis followed by Dunn’s post-test. Data are presented as mean ± standard error of the mean (SEM).

## 5. Conclusions

This work analyzed, for the first time, a derivative of curcumin, EF24, in SW13 and H295R cell lines. Curcumin and its derivatives have been explored in different tumor types, and this work adds a new piece to the hard fight against cancer. Undeniably, much effort is still needed for the future use of these compounds for humans. Nonetheless, the research has to start from preclinical work, and indeed, this study has suggested an anti-tumor effect of EF24 in adrenocortical tumor cell lines.

## Supplementary Materials

The following are available online, Figure S1: Western blot quantitative densitometry for SW13 and H295R cells treated for 24 h and 72 h respectively. (A) SW13 cells; (B) H295R cells. C: control. E: EF24 at 6.5 μM for SW13 and 5 μM for H295R cells. E+M: EF24+mitotane. M: mitotane 8 μM for SW13 and 10 μM for H295R cells. Comparison of phosphorylated proteins expression as a ratio normalized to untreated control levels. Treatment vs control: * *p* < 0.05; Figure S2: Representative flow cytometric analysis of intracellular ROS levels in SW13 and H295R cells treated for 24 h and 72 h respectively, with EF24, mitotane or their combination. DCFH-DA= 2’,7’-dichlorofluorescein diacetate. NAC = N-acetil-cysteine. E = EF24. M = mitotane. E + M = EF24 + mitotane.
