# Supplementary material for: Biological Effects of EF24, a Curcumin Derivative, Alone or Combined with Mitotane in Adrenocortical Tumor Cell Lines
Source: Molecules. 2019 Jun 12;24(12):2202. doi: 10.3390/molecules24122202 (PMC6630722; doi:10.3390/molecules24122202)
Supplement: Supplementary file 1 [file molecules-24-02202-s001.pdf]

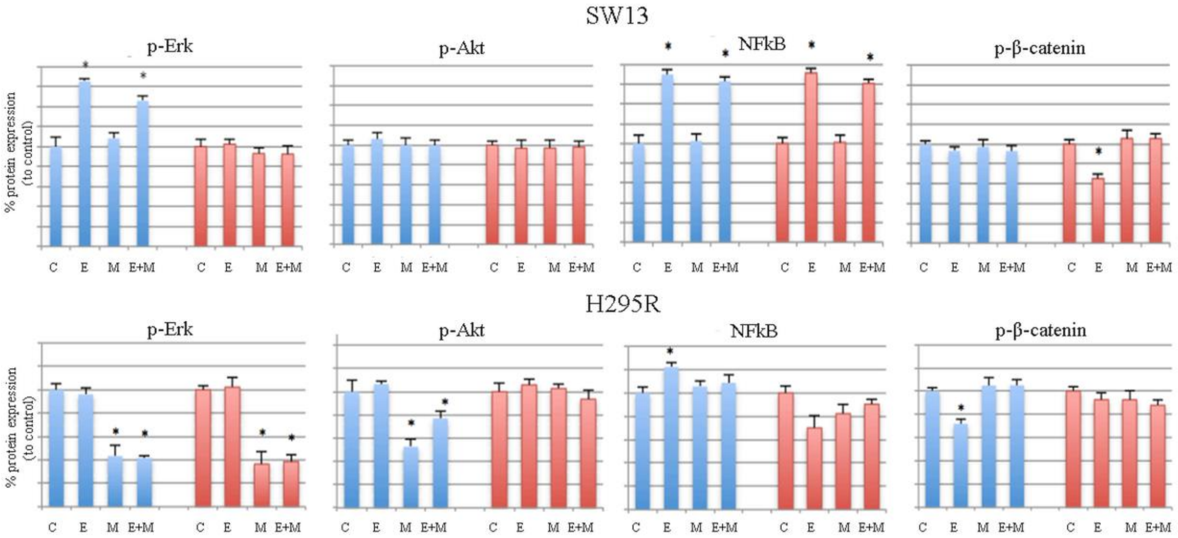

Figure 1. Western blot quantitative densitometry for SW13 and H295R cells treated for 24h and 72h respectively. A) SW13 cells; B) H295R cells. C: control. E: EF24 at 6.5 μM for SW13 and 5 μM for H295R cells. E+M: EF24+mitotane. M: mitotane 8 μM for SW13 and 10 μM for H295R cells. Comparison of phosphorylated proteins expression as a ratio normalized to untreated control levels. Treatment vs control: * $P<0.05$.

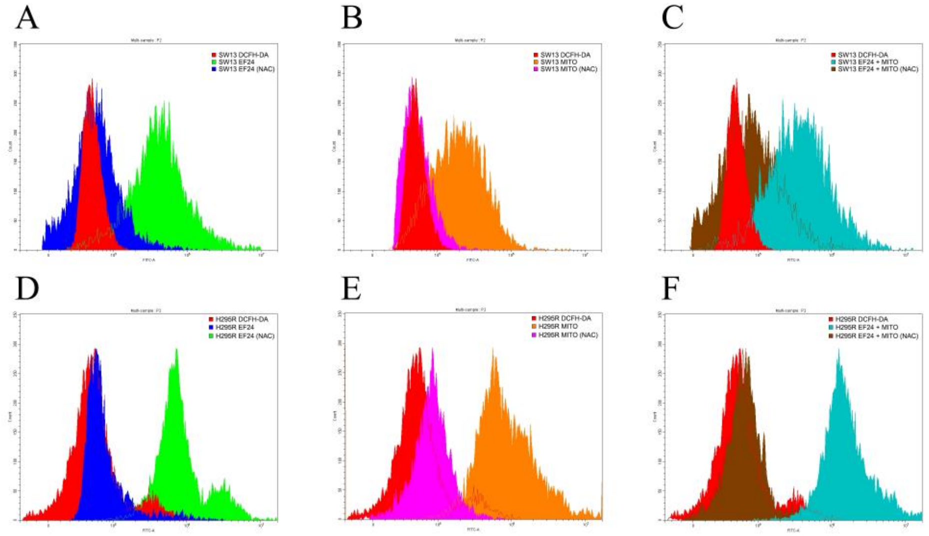

Figure 2. Representative flow cytometric analysis of intracellular ROS levels in SW13 and H295R cells treated for 24h and 72h respectively, with EF24, mitotane or their combination. DCFH-DA= 2′,7′-dichlorofluorescein diacetate. NAC= N-acetil-cysteine. E=EF24. M=mitotane. E+M=EF24+mitotane.
